# Demethylation of the NRF2 Promoter Protects Against Carcinogenesis Induced by Nano-SiO_2_

**DOI:** 10.3389/fgene.2020.00818

**Published:** 2020-07-28

**Authors:** Dan Lou, Xiaoyi Wei, Ping Xiao, Qian Huo, Xinyu Hong, Jingqiu Sun, Yi Shuai, Gonghua Tao

**Affiliations:** ^1^Shanghai Municipal Center for Disease Control & Prevention, Shanghai, China; ^2^Department of Environmental Health and Engineering, Bloomberg School of Public Health, Johns Hopkins University, Baltimore, MD, United States; ^3^Department of Food Science, Shanghai Business School, Shanghai, China; ^4^Syngenta (China) Investment Company Limited, Shanghai, China

**Keywords:** Nano-SiO_2_, malignant transformation, carcinogenesis, DNA methylation, NRF2

## Abstract

Nano silicon dioxide (Nano-SiO_2_) has been widely used in industries such as the field of biomedical engineering. Despite the existing evidence that Nano-SiO_2_ exposure could induce oxidative stress and inflammatory responses in multiple organ systems, the carcinogenicity of Nano-SiO_2_ exposure has rarely been investigated. Thus in this study, two types of human bronchial epithelial cell lines (16HBE and BEAS-2B) were selected as *in vitro* models to investigate the carcinogenicity of Nano-SiO_2_. Our results revealed that Nano-SiO_2_ induces a malignant cellular transformation in human bronchial epithelial cells according to the soft agar colony formation assay. The carcinogenesis induced by Nano-SiO_2_ was also confirmed in nude mice. By using immunofluorescence assay and high-performance capillary electrophoresis (HPCE), we observed a genome-wide DNA hypomethylation induced by Nano-SiO_2_. Besides the reduced enzyme activity of total DNMTs upon Nano-SiO_2_ treatment, altered expression of DNMTs and methyl-CpG binding proteins were observed. Besides, we found that the expression of NRF2 was activated by demethylation of CpG islands within the NRF2 promoter region and the overexpression of NRF2 could alleviate the carcinogenesis induced by Nano-SiO_2_. Taken together, our results suggested that Nano-SiO_2_ induces malignant cellular transformation with a global DNA hypomethylation, and the demethylation of NRF2 promoter activates the expression of NRF2, which plays an important role in protecting against the carcinogenesis induced by Nano-SiO_2_.

## Introduction

Nano silicon dioxide (Silica nanoparticles, Nano-SiO_2_), due to its special characteristics of large surface area and optical transparency, has been widely used in various fields including biomedical imaging, drug delivery, cosmetics, and electronics industry (Vivero-Escoto et al., 2012; Sweeney et al., 2016; Mohajerani et al., 2019). Meanwhile, its widespread applications raise potential health risks to humans through occupational and environmental exposure. Recent studies have demonstrated that Nano-SiO_2_ exposure in a short term could induce oxidative stress, mitochondria dysfunction, and inflammatory responses in multiple organ systems (Song et al., 2009; Napierska et al., 2010; Fruijtier-Polloth, 2012; Lu et al., 2013), among which respiratory system is the primary site that exposed to the airborne Nano-SiO_2_ particles (Song et al., 2009; Yu et al., 2015).

Long-lasting nanomaterials in certain tissues have been reported to cause chronic adverse effects such as carcinogenesis due to their special physicochemical properties (Hirose et al., 2011), and nano-sized particles might be more carcinogenic than micron-sized particles concerning long-term exposure (Gebel, 2012). Inhalation of TiO_2_ nanoparticles was shown to increase the risk of lung cancers in rats (Bermudez et al., 2004) and carbon nanotube exposure through inhalation is carcinogenic to the lungs of male and female rats (Kasai et al., 2016). Although SiO_2_ has been classified as carcinogenic to humans by the International Agency for Research on Cancer (IARC) in 1997 (Iarc., 1997), there is limited data on carcinogenicity following chronic exposure to Nano-SiO_2_. Nano-SiO_2_ can be localized to the nucleus thereby affecting nuclear integrity and causing DNA damage (Chen and von Mikecz, 2005; Wang et al., 2007).

Alterations in DNA methylation, a typical epigenetic modification, have been reported in cells exposed to Nano-SiO_2_. A global genomic hypomethylation induced by Nano-SiO_2_ was observed in HaCaT cells and the CpGs in the promoter of PARP1 gene were demethylated, resulting in DNA repair dysfunction (Gong et al., 2010, 2012b). Abnormal DNA methylation occurs frequently in the process of carcinogenesis (Miousse et al., 2018; Dreval et al., 2019), through devastating genome stability and activating aberrant transcription (Gama-Sosa et al., 1983). Decreased global DNA methylation and increased expression of DNA methyltransferases (DNMTs) were demonstrated in the early stage of Nano-SiO_2_-induced malignant cellular transformation (Seidel et al., 2017), indicating that DNA methylation is involved in the Nano-SiO_2_-induced carcinogenesis.

Nuclear factor erythroid-2-related factor 2 (NRF2) is a key transcription factor involved in cellular responses to stresses induced by electrophiles, oxidants, and chemicals (Motohashi and Yamamoto, 2004). In addition to stress responses, NRF2 plays a contradictory role in cancers (Wu et al., 2019). Loss of NRF2 ultimately leads to malignant cellular transformation in the prostate gland of murine models (Frohlich et al., 2008). NRF2 dysfunction in mice accelerates the acetylhydrolase-induced hepatocarcinogenesis (Marhenke et al., 2008). On the contrary, accumulating evidence indicated that increased activation of NRF2 frequently occurs in multiple types of tumors, which promotes cancer cell growth and metastasis formation (Liu et al., 2016; Zhang et al., 2016; Lu et al., 2017). The hypomethylation of CpG islands within the NRF2 promoter region was reported in colorectal cancer (Kang et al., 2014; Zhao et al., 2015). Decreased NRF2 expression due to hypermethylation of CpG islands within the NRF2 promoter region was found to be associated with prostate cancer (Yu et al., 2010; Khor et al., 2014).

In the present study, we demonstrated that Nano-SiO_2_ induces a malignant cellular transformation and a global DNA hypomethylation in human bronchial epithelial cells. Intriguingly, we found that demethylation of CpG islands within the NRF2 promoter region increases the NRF2 gene expression, which inhibits the carcinogenesis induced by Nano-SiO_2_.

## Materials and Methods

### Chemicals and Reagents

The 15 nm Nano-SiO_2_ particles were purchased from Wan Jing New Material Co., Ltd. (Hangzhou, Zhejiang, China) and were characterized as previously described (Gong et al., 2012a). Nano-SiO_2_ samples were sonicated to distribute in the solution as evenly as possible. Dosing solutions were prepared by dissolving the calculated amount of Nano-SiO_2_ in the cell culture medium, following the approved standard operating procedures for handling toxic agents. All other reagents were obtained from commercial sources and were of the highest available grade.

### Cell Culture

Human bronchial epithelial cell lines (16HBE and BEAS-2B) and adenocarcinomic human alveolar basal epithelial cell line (A549) were purchased from the Cell Bank in Chinese Academy of Sciences Cell Bank in Shanghai. Frozen cells were thawed and expanded in MEM medium supplemented with 10% fetal bovine serum (FBS), 10 U/ml penicillin, and 10 U/ml streptomycin. Cells were incubated in a humidified atmosphere with 5% CO_2_ at 37°C and passaged at about 80% confluence.

### Nano-SiO_2_ Treatment

Human bronchial epithelial cell lines were treated with Nano-SiO_2_ for several passages and harvested at certain time points as shown in Figure 2A. In detail, 16HBE and BEAS-2B cells were grown in complete MEM medium until they reached a confluence of about 70–80%. Cells were then treated with Nano-SiO_2_ (10.0 μg/ml for 16HBE cells, 40.0 μg/ml for BEAS-2B cells) for 24 h and changed into the complete MEM medium until they are ready for subculture, these cells treated with Nano-SiO_2_ for one passage are referred to P1. Accordingly, cells treated as above for n passages are named as P(n). Cells cultured in complete MEM medium for the same passages without any Nano-SiO_2_ treatment are taken as the negative control. A549, an epithelial cell line derived from human lung carcinoma, was used as a positive control.

### Cell Viability Measurement by MTT Assay

Cell viability was determined by MTT assay. 16HBE and BEAS-2B cells growing at the exponential phase were seeded in 96-well plates with a density of 5 × 10^4^cells/ml. After treated with various dosages of Nano-SiO_2_ (0, 1.0, 2.5, 5.0, 10.0, 25.0, and 50.0 μg/ml for 16HBE, 0, 1.0, 5.0, 10.0, 20.0, 40.0, 80.0, and 160.0 μg/ml for BEAS-2B) for 24 h, 50 μl MTT solution was added to each well and incubated for another 4 h. After adding 150 μl DMSO, the absorbance of each well was measured at 490 nm using a spectrophotometer. Each treatment group has at least three replicates. Cell viability was obtained as a percentage of the value of viable cells in the control groups.

### Anchorage-Independent Cell Growth Measured by Soft Agar Colony Formation Assay

Anchorage-independent growth of Nano-SiO_2_-treated human bronchial epithelial cells (16HBE and BEAS-2B) was measured by soft agar colony formation assay. Cells treated with Nano-SiO_2_ for various passages (P0, P8, P16, and P32 of 16HBE cells, P0, P15, P30, and P45 of BEAS-2B cells) were trypsinized. Cells were resuspended at a density of 1 × 10^4^/ml in MEM medium with 0.3% agar and plated over 3 ml of a solidified complete MEM culture medium containing 0.5% agar. Cells were then incubated in a humidified atmosphere with 5% CO_2_ at 37°C for 3 weeks. The colonies were photographed and counted by using ImageJ software.

### Tumorigenicity in Nude Mice

The tumorigenicity of the Nano-SiO_2_-treated human bronchial epithelial cells (16HBE and BEAS-2B) was determined in nude mice. Balb/c-nu male nude mice (4 weeks old) purchased from the Laboratory Animal Center in Shanghai, were housed in ventilated microisolators with sterile food and water. After one week of acclimation, mice were randomly divided into eight treatment groups, with three mice in each group. Specifically, four groups of mice were injected with different passages of 16HBE cells (P0, P8, P16, and P32), another four groups of mice were injected with different passages of BEAS-2B cells (P0, P15, P30, and P45). Nude mice injected with cells cultured in complete MEM medium for the same passages without any Nano-SiO2 treatment were taken as the negative control. Cells were harvested by trypsinization, washed, and resuspended in PBS. Cell suspensions were then injected (0.2 ml) subcutaneously to mice at a density of 1 × 10^7^ cells/ml. Tumor growth was monitored for 90 consecutive days after injection by measuring the major diameter of the tumor externally with a slide caliper. Mice were sacrificed by cervical dislocation if the major diameter of its tumor reaches about 2 cm. Mice were kept for more than 90 days after injection. The tumor sizes were measured at the fifth week after injection and the incidence and latency period of tumorigenesis were recorded. The incidence was presented as the number of mice with a tumor at the site of injection versus the total number of mice. The latency period is defined as the time required for a tumor to be able to visually detect.

### Quantitative Reverse Transcription-PCR (qRT-PCR) Assay

Total RNAs from three cell lines (16HBE, BEAS-2B, and A549) were isolated and reverse transcribed into cDNAs by using the PrimeScript^TM^ RT reagent kit with gDNA Eraser (Takara Bio Inc., Japan). Four genes related to the NRF2 signaling pathway were selected for qRT-PCR assay. Primers are listed in Supplementary Table S1. All qPCR reactions were performed on an Applied Biosystems^TM^ StepOne^TM^ Real-time PCR system using iTaq^TM^ Universal SYBR Green Supermix, with three technical replicates. The amplification procedure was as follows: 95°C for 5 min, followed by 40 cycles of 95°C for 10 s and 60°C for 20 s. Relative quantification of target genes was performed using the ΔΔCt method with GAPDH as a reference gene.

### Western Blotting Assay

Cells were washed with ice-cold PBS and lysed on ice with a protease inhibitor cocktail. Protein concentrations were measured by BCA method. Protein samples were separated by sodium dodecyl sulfate-polyacrylamide gel electrophoresis (SDS-PAGE) and transferred to PVDF membranes. The membranes were probed with NRF2 (1:500), HO1 (1:1000), SOD1 (1:1000), GST (1:1000), DNMT1 (1:500), DNMT3a (1:500), DNMT3b (1:500), MeCP2 (1:500), MBD2 (1:500), GAPDH (1:10000) antibodies (Santa Cruz Biotechnology, Inc., Texas, United States) at 4°C overnight. The bands were visualized after incubation with a chemiluminescent substrate. Quantification of the band density was determined by densitometric analysis.

### 5-mC Content Measurement by Immunofluorescence Assay

Cells were fixed with 4% paraformaldehyde for 15 min and then fixed with cold formaldehyde for 5 min, washed with PBST, and then incubated with hydrochloric acid at 37°C for 30 min. After blocked with 3% PBST-BSA for 30 min, samples were incubated with anti-5-mC antibody (1:1000) at 37°C for 1 h and incubated with FITC-conjugated secondary antibody (1:400) for 30 min. Finally, coverslips were then incubated with DAPI for double staining and then mounted on glass slides by using Fluo-Antifading medium II. The fluorescence intensity was detected by using an inverted fluorescent microscope.

### Measurement of DNMT Enzymes Activity

Cells treated with Nano-SiO_2_ for different passages (P0, P8, P16, and P32 of 16HBE cells, P0, P15, P30, and P45 of BEAS-2B cells) and A549 cells were harvested by trypsinization. Nuclear was extracted and total DNMT enzymes activity was determined by EpiQuik^TM^ DNA Methyltransferase Activity/Inhibition Assay Kit (EpiGentek, United States) according to the procedures provided by the manufacturers.

### Qualitative Analysis of DNA Methylation by MSP

Genomic DNA was extracted and treated with bisulfite as previously described (Gong et al., 2012a). After sodium bisulfite conversion, PCR assay was conducted to analyze the DNA methylation level in specific *NRF2* gene loci quantitatively. Primer sets M1 and M2 were designed and synthesized to amplify the methylated DNA, primer sets U1 and U2 were designed and synthesized to amplify the unmethylated DNA. PCR products were separated by electrophoresis at 100 V for 40 min on 2.2% agarose gels. Primers were listed in Supplementary Table S1.

### Cell Transfection

The cells (16HBE and BEAS-2B) were separately transfected with NRF2 shRNA(h) lentiviral particles (sc-37030-V) or plasmid pEGFP-NRF2 to construct NRF-2 knockdown (KD) or overexpression (OE) cell lines, according to the manufacturer’s instructions. For the knockdown of NRF-2, NRF2 shRNA(h) lentiviral particles (shRNA-NRF2) and control shRNA Lentiviral Particles (shRNA-Ctrl) were transfected into cells. After transfected for 24 h, the transfection culture medium was replaced with complete culture medium and incubated for 72 h. Then, 10 μg/ml puromycin dihydrochloride was used to select and achieve stable knockdown cell lines. For the overexpression of NRF-2, pEGFP containing NRF-2 gene sequence (pEGFP-NRF2) or empty pEGFP (pEGFP) were transfected into cells. Full length cDNAs of NRF-2 were amplified through RT-PCR using specific forward 5′-CACCATGGGAATGGACTTGGAGCTGCC-3′ and reverse 5′-CTAGTTTTTCTTAACATCTGGCTTCTTAC-3′ primers. After transfected for 24 h, cells were selected by G418. Selected cells were harvested for transfection efficiency confirmation and subsequent experiments (Supplementary Figure S1).

### Statistical Analysis

Data were represented by the means ± SD of at least three independent experiments. Statistical significances among experimental groups were evaluated by ANOVA followed by the Tukey *post hoc* test performed with the GraphPad Prism (version 8.0.2; San Diego, CA, United States).

## Results

### Nano-SiO_2_ Reduces Cell Viability of Human Bronchial Epithelial Cells

**FIGURE 1:**
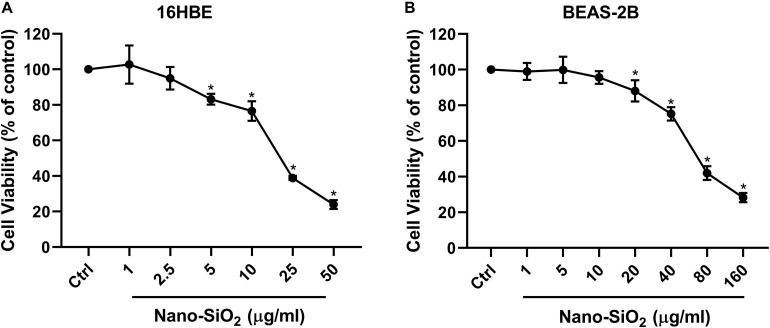
Cell viability of two types of human bronchial epithelial cells **(A:** 16HBE cell line, **B:** BEAS-2B cell line) upon treatment with Nano-SiO_2_ for 24 h. Values were mean ± SD (*n* = 3); *p < 0.05, control versus Nano-SiO_2_ treatment.

Since the respiratory system is the primary site that exposed to the airborne Nano-SiO_2_ particles (Song et al., 2009; Yu et al., 2015), two types of human bronchial epithelial cell lines (16HBE and BEAS-2B) were selected as our *in vitro* models. To investigate the cytotoxicity of Nano-SiO_2_, we determined the effects of Nano-SiO_2_ on the viability of human bronchial epithelial cells by MTT assay. The survival rate of cells treated with Nano-SiO_2_ was expressed as the percentage of that of cells in the control group without nano-SiO_2_ treatment. For 16HBE cells, no significant change in cell viability was observed when treated with 1.0 or 2.5 μg/ml Nano-SiO_2_ for 24 h. After exposure to 10.0 μg/ml Nano-SiO_2_ for 24 h, cell viability was reduced to 77.04% of the control group (*p* < 0.05) (Figure 1A). For BEAS-2B cells, no significant change in cell viability was observed when treated with 1.0, 5.0, or 10.0 μg/ml Nano-SiO_2_ for 24 h (Figure 1B). Cell viability was significantly decreased when treated with Nano-SiO_2_ no less than 20.0 μg/ml. At a concentration of 40.0 μg/ml Nano-SiO_2_, cell viability was reduced to 75.18% of the control group after 24 h (*p* < 0.05) (Figure 1B).

With the increase of Nano-SiO_2_ concentration, the reduction of cell viability in both cell lines became more severe, showing a dose-dependent manner. Based on the cell viability results, 10 μg/ml and 40 μg/ml Nano-SiO_2_ were used as the treatment dosage in subsequent experiments for 16HBE and BEAS-2B cells, respectively.

### Nano-SiO_2_ Induces Malignant Transformation of Human Bronchial Epithelial Cells

To confirm the malignant cellular transformation induced by Nano-SiO_2_, a soft agar colony formation assay was performed to evaluate the ability of Nano-SiO_2_-treated cells to grow independently on a solid surface (anchorage-independent growth). The number of colonies grown from 16HBE cells cultured in complete MEM medium (control) for 8, 16, and 32 passages without Nano-SiO_2_ exposure was first compared, and no obvious difference was observed (data not shown). Similarly, no obvious difference in the numbers of the colony was observed among the 15th, 30th, and 45th passage of BEAS-2B cells in the control group (data not shown). Hereafter, when compared with treatment groups, the control group was named as 16HBE-P0 or BEAS-2B-P0.

**FIGURE 2:**
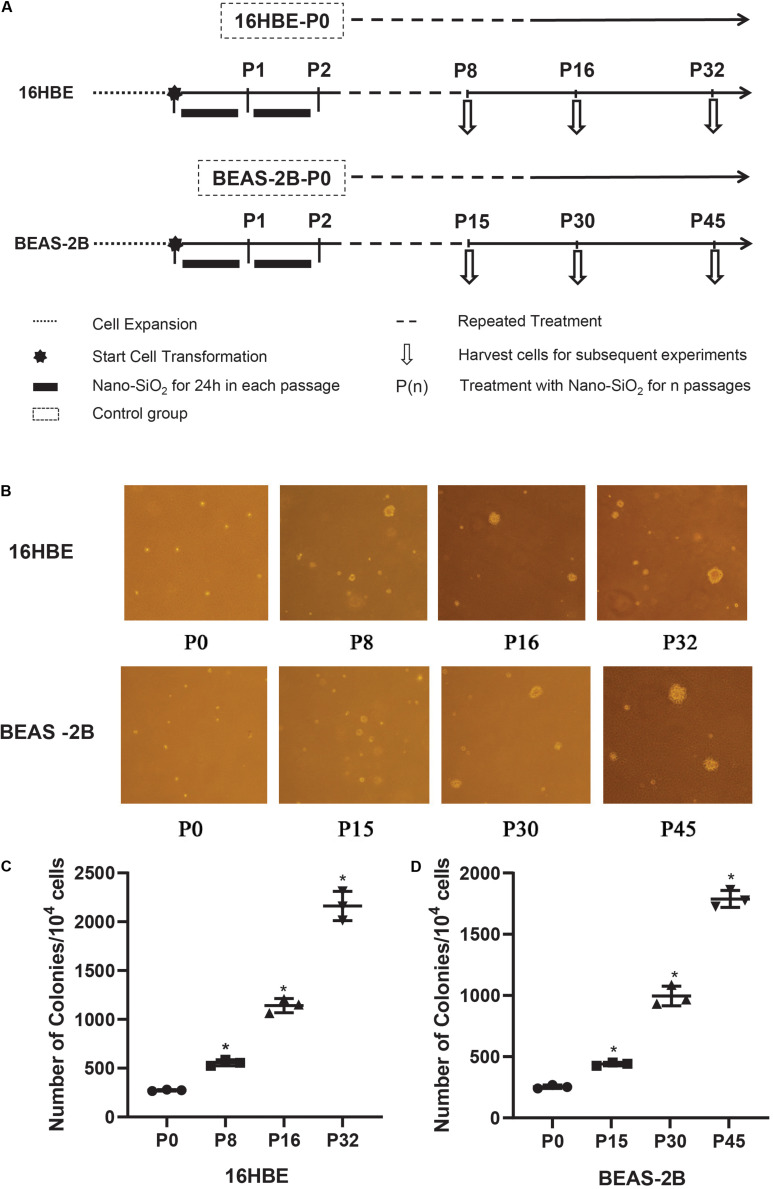
The effects of Nano-SiO_2_ on soft agar colony formation of 16HBE and BEAS-2B cells. **(A)** The experimental protocol indicates the Nano-SiO_2_ treatment timeline of 16HBE and BEAS-2B cells. **(B)** Representative colonies on soft agar plates. Cells treated with Nano-SiO_2_ (10 μg/ml for 16HBE cells, and 40 μg/ml for BEAS-2B cells) or without Nano-SiO_2_ (16HBE-P0, BEAS-2B-P0) were grown on soft agar plates after indicated passages. **(C,D)** Dot plots showing the number of colonies developed from 16HBE and BEAS-2B cells. The data are shown as mean ± SD (*n* = 3); *p < 0.05, control (P0) vs. Nano-SiO_2_ treatment.

We observed that with the prolonged exposure to Nano-SiO_2_, both 16HBE and BEAS-2B cells developed a significant amount of colonies in the soft agar (Figure 2B). Specifically, after treated with 10 μg/ml Nano-SiO_2_ for 8 passages, ten thousand 16HBE cells developed 555 colonies in the soft agar, which is approximately twice that from 16HBE-P0 cells which are not exposed to Nano-SiO_2_. As the Nano-SiO_2_ exposure continued for 32 passages, ten thousand 16HBE cells developed 2161 colonies, which is eight times as many as those growing from 16HBE-P0 cells (Figure 2C).

Similarly, after treated with 40 μg/ml Nano-SiO_2_ for 15 passages, ten thousand BEAS-2B cells developed 440 colonies, which is approximately twice as many as the colonies growing from BEAS-2B-P0 cells. As the Nano-SiO_2_ exposure continued for 45 passages, ten thousand BEAS-2B cells developed 1788 colonies, which is seven times as many as the colonies growing from BEAS-2B-P0 cells (Figure 2D). The results indicated that Nano-SiO_2_ could induce malignant cell transformation in human bronchial epithelial cells and promote tumorigenesis *in vitro*.

### Tumorigenicity of Nano-SiO_2_-Transformed Cells in Nude Mice

To assess the *in vivo* carcinogenicity of Nano-SiO_2_-treated cells, twenty-four nude mice received subcutaneous injections of human bronchial epithelial cells with various treatments (three mice in each group). The features of the tumor formed at the site of cell injection are summarized in Table 1. We did not observe any tumor when 16HBE or BEAS-2B cells were not exposed to Nano-SiO_2_ (P0). In contrast, both 16HBE and BEAS-2B cells treated with Nano-SiO_2_ formed tumors in nude mice within five weeks. The latent period of tumorigenesis induced by cells treated with Nano-SiO_2_ for a longer period was generally shorter than cells treated for a shorter period. Also, the size of tumors growing in nude mice correlated with the duration of Nano-SiO_2_ treatment. Specifically, at the fifth week after injection, P8-, P16-, and P32- 16HBE cells formed tumors with a mean diameter of 2, 4, and 7 mm, respectively. Similarly, at the fifth week after injection, P15-, P30-, and P45- BEAS-2B cells formed tumors with a mean diameter of 3, 4, and 8 mm, respectively. Cells treated with Nano-SiO_2_ for a longer time tend to induce larger tumors in nude mice. These results indicated that human bronchial epithelial cells could be malignantly transformed by Nano-SiO_2_ exposure and acquire tumorigenicity *in vivo*.

**TABLE 1:** Tumorigenicity in nude mice of 16HBE and BEAS-2B cells treated with Nano-SiO_2_.

		**Tumors formed at the injection site**		
**Cell lines**	**Treatment group**	**Incidence^a^**	**Latency period^b^ (weeks)**	**Tumor size (mm)^c^ Diameter mean (range)**
16HBE	P0	0/3	NA	NA
	P8	3/3	5	2 (2–3)
	P16	3/3	3	4 (3–6)
	P32	3/3	1	7 (6–9)
BEAS-2B	P0	0/3	NA	NA
	P15	3/3	5	3 (2–4)
	P30	3/3	3	4 (4–6)
	P45	3/3	2	8 (6–9)

*^*a*^The incidence was presented as the number of mice with a tumor at the site of injection versus the total number of mice. ^*b*^The latency period is the time required for a tumor to be able to visually detect. No tumor regression was ever observed. ^*c*^The tumor sizes were measured at the 5th week after injection.*

### Nano-SiO_2_ Induces Global DNA Hypomethylation in Human Bronchial Epithelial Cells

Since global changes in DNA methylation are a hallmark of carcinogenesis, the levels of genome-wide DNA methylation in Nano-SiO_2_ treated cells were determined. Immunofluorescence assay was first performed to estimate the relative intensity of 5-mC in human bronchial epithelial cells (16HBE and BEAS-2B cells) and A549 cells (human lung carcinoma cell line, as a positive control).

**FIGURE 3:**
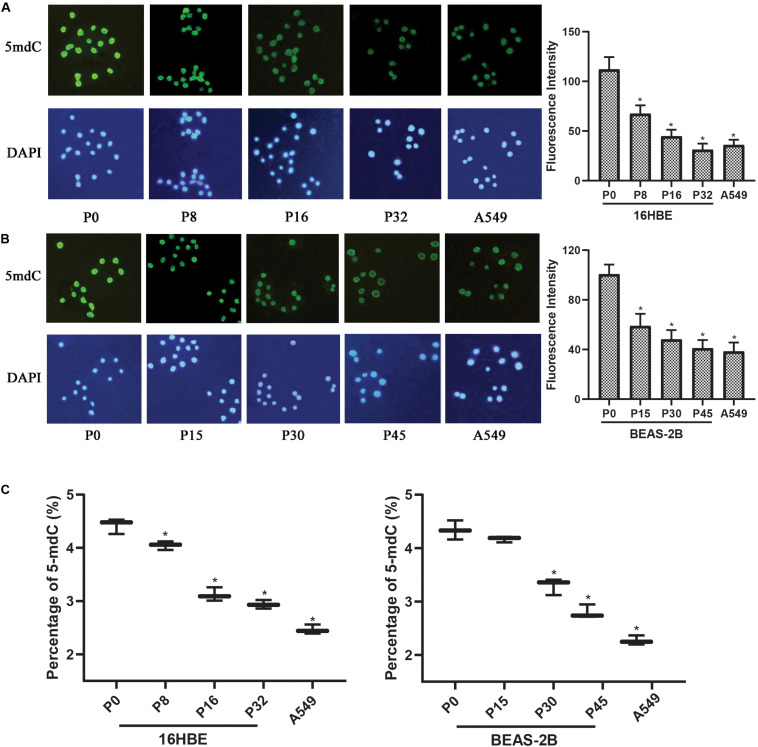
Nano-SiO_2_ exposure induces global DNA hypomethylation in human bronchial epithelial cells. 16HBE cells were treated with 10 μg/ml Nano-SiO_2_ for 8, 16, or 32 passages and BEAS-2B cells were treated with 40 μg/ml Nano-SiO_2_ for 15, 30, or 45 passages. **(A,B)** Immunofluorescence assay was performed to detect 5-mC levels in 16HBE and BEAS-2B cells. 5-mC was visualized by immunostaining with the antibodies against 5-mC (green) and nuclei were visualized by staining with DAPI (blue). Quantification of relative fluorescence intensity was shown on the right. **(C)** The levels of 5-mdC normalized to that of cytosine were shown in percentage. The data are presented as mean ± SD (*n* = 3). *p < 0.05, compared with the control group (P0). A549, an epithelial cell line derived from human lung carcinoma, was used as a positive control.

A significant decrease in the fluorescence intensity of 5-mC was observed in both 16HBE and BEAS-2B cells treated with Nano-SiO_2_. For 16HBE cells, the mean fluorescence intensity of 5-mC was reduced significantly by 39.85%, 60.14%, and 72.18% in P8, P16, and P32 cells, respectively, when compared with the control group (16HBE-P0) (Figure 3A). For BEAS-2B cells, the mean fluorescence intensity of 5-mC was reduced significantly by 41.40%, 52.18%, and 59.23% in P15, P30, and P45 cells, respectively, when compared with the control group (BEAS-2B-P0) (Figure 3B).

High-performance capillary electrophoresis (HPCE) assays were performed to further investigate the levels of genome DNA methylation in cells treated with Nano-SiO_2_. We found that the levels of 5-mdC were reduced in cells exposed to Nano-SiO_2_ in both 16HBE and BEAS-2B cells when compared with the non-exposed cells in P0, the control group (Figure 3C). Besides, the extent of 5-mdC reduction correlated with the duration of Nano-SiO_2_ exposure (Figure 3C). These results suggested that Nano-SiO_2_ exposure induces genome-wide DNA hypomethylation of human bronchial epithelial cells, and the decrease of DNA methylation is aggravated with prolonged Nano-SiO_2_ exposure.

### Influences of Nano-SiO_2_ on the Expression of DNA Methylation-Associated Proteins

**FIGURE 4:**
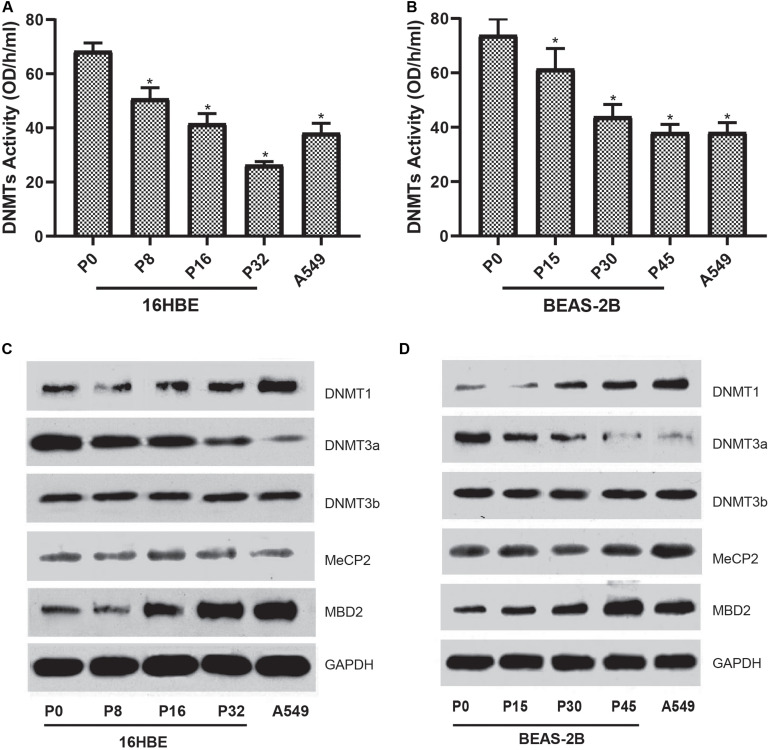
Effects of Nano-SiO_2_ on the expression of DNA methylation-associated proteins in human bronchial epithelial cells. DNMTs activity of 16HBE cells **(A)** and BEAS-2B cells **(B)** treated with Nano-SiO_2_ for various durations were determined. The data are shown as mean ± SD (*n* = 3). *p < 0.05, compared with the control group (P0). A549, an epithelial cell line derived from human lung carcinoma, was used as a positive control. Levels of DNMT-1, DNMT-3a, DNMT-3b, MeCP-2, and MBD-2 in 16HBE cells **(C)** and BEAS-2B cells **(D)** treated with Nano-SiO2 for different passages were evaluated using western blot. GAPDH was used as the loading control.

To characterize the global DNA hypomethylation in cells treated with Nano-SiO_2_, the total activity of DNA methyltransferases (DNMTs) catalyzing DNA methylation was investigated. We found that the total activity of DNMTs in both cell lines (16HBE and BEAS-2B) was gradually inhibited with the prolonged treatment with Nano-SiO_2_ (Figures 4A,B). We further determined the levels of DNMTs in cells treated with Nano-SiO_2_. According to the results of western blot analysis, we found that the levels of DNMT1 are increased while DNMT3a protein expression is decreased in cells treated with Nano-SiO_2_ (Figures 4C,D). Further investigation on levels of methyl CpG binding proteins (MeCP-2 and MBD2) revealed that Nano-SiO_2_ exposure significantly increases MBD2 protein expression in both 16HBE and BEAS-2B cells. No significant change was observed in levels of MeCP-2 in both cell lines after Nano-SiO_2_ treatment (Figures 4C,D).

### Nano-SiO_2_ Inhibits the CpG Methylation at the Promoter Region of NRF2 Gene

**FIGURE 5:**
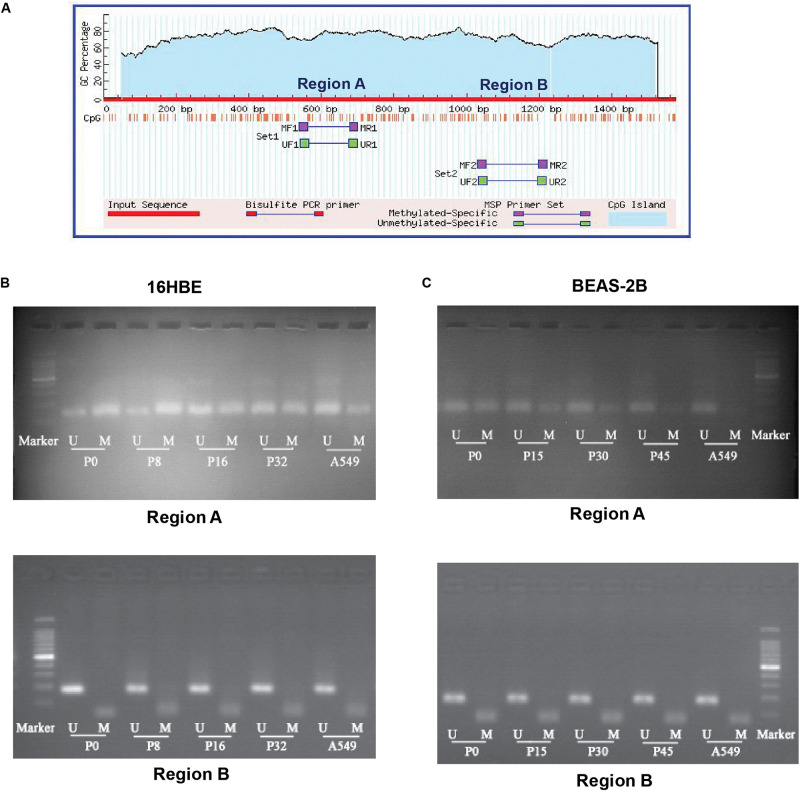
MSP analysis of DNA methylation of the promoter of the *NRF2* gene. **(A)** Schematic graph showing CpG islands in the *NRF2* promoter region and binding sites for MSP PCR primers. **(B,C)** Representative electrophoresis results of PCR products of CpG islands in the *NRF2* promoter region using primers illustrated in **(A)**. M: methylated alleles. U: unmethylated alleles. A549 was used as a positive control.

The transcription factor NRF2 involved in stress response plays a role in cancer development. Expression of the NRF2 gene is known to be influenced by the status of methylation of CpG islands in the promoter region in cancer cells (Kang et al., 2014; Zhao et al., 2015). Therefore, we asked whether DNA methylation of the promoter region of the NRF2 gene is influenced by Nano-SiO_2_ exposure. We conducted a methylation-specific PCR (MSP) to assess the methylation status of CpG sites at the NRF2 promoter region. As illustrated in Figure 5A, two sets of primers (Supplementary Table S2) were designed to detect the methylation status in different CpG islands (region A and region B). The methylation level at region A of 16HBE cells treated with Nano-SiO_2_ for 16 (P16) and 32 (P32) passages were decreased when compared with the untreated group (16HBE-P0). Similarly, the methylation level at region A of BEAS-2B cells treated with Nano-SiO_2_ for 15, 30, and 45 passages was also reduced when compared with the untreated group (BEAS-2B-P0). However, no obvious changes were found at region B in 16HBE or BEAS-2B cells after Nano-SiO_2_ treatment (Figures 5B,C).

### Expression of the NRF2 Gene Is Upregulated in Cells Exposed to Nano-SiO_2_

**FIGURE 6:**
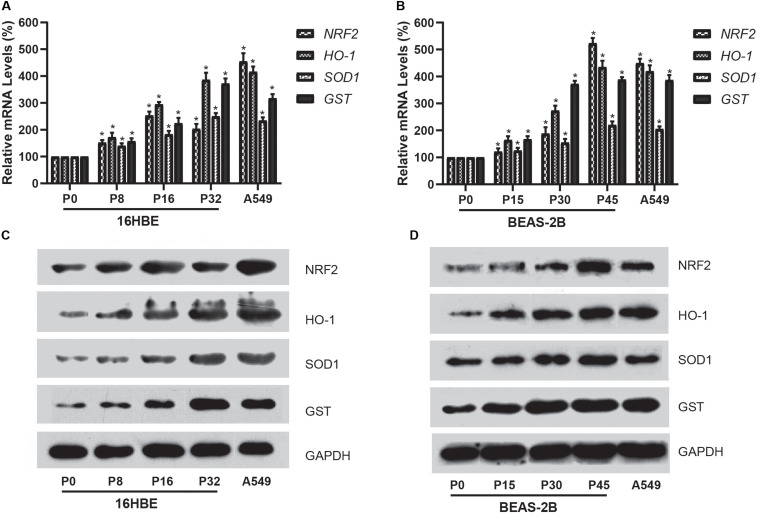
Effects of Nano-SiO_2_ on the expression of *NRF2* and its target genes in human bronchial epithelial cells. **(A,B)** mRNA expression of NRF2, HO-1, SOD1, and GST genes in 16HBE or BEAS-2B cells were determined by qRT-PCR. **(C,D)** Protein expression levels of NRF2, HO-1, SOD1, and GST in 16HBE or BEAS-2B cells were evaluated by western blotting analysis. GAPDH was used as the internal control for normalization. The data are presented as mean ± SD (*n* = 3). *p < 0.05, compared with the untreated control group (P0). A549 was used as a positive control.

We further investigated the expression of NRF2 gene using qRT-PCR for mRNA and western blot analysis for protein in cells treated with Nano-SiO_2_. Our results showed that Nano-SiO_2_ exposure significantly increases the mRNA and protein expression of the NRF2 gene in both cell lines (Figures 6A–D), which is consistent with the hypomethylation of global DNA induced by Nano-SiO_2_ (Figures 3A,C). Furthermore, we also determined the expression levels of several gene targets (HO-1, SOD1, and GST) of the transcription factor NRF2 upon Nano-SiO_2_ exposure. Analysis using qPCR and western blot showed that the mRNA and protein levels of HO-1, SOD1, and GST were also significantly increased by Nano-SiO_2_ treatment. The mRNA and protein expression of these anti-oxidative genes regulated by NRF2 largely correlated with the duration of Nano-SiO_2_ exposure (Figure 5). These results revealed that the NRF2 gene and its target genes were up-regulated by Nano-SiO_2_ exposure.

### NRF2 Inhibits the Carcinogenesis of Human Bronchial Epithelial Cells Induced by Nano-SiO_2_

**FIGURE 7:**
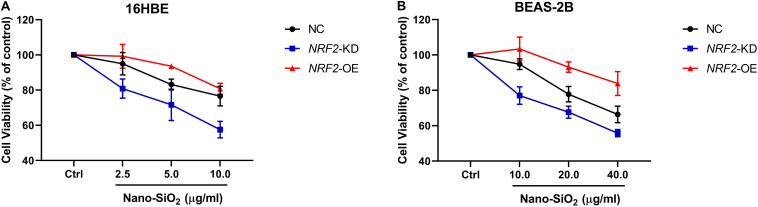
The NRF2 protein protects against the cytotoxicity of Nano-SiO_2_ on human bronchial epithelial cells. Cells **(A)** 16HBE, **(B)** BEAS-2B) were treated with Nano-SiO_2_ particles for 24 h. Values were mean ± SD (*n* = 3). NC: cells (16HBE or BEAS-2B) transfected with negative control; NRF2-KD: knockdown of the NRF2 gene; NRF2-OE: NRF2 overexpression.

**FIGURE 8:**
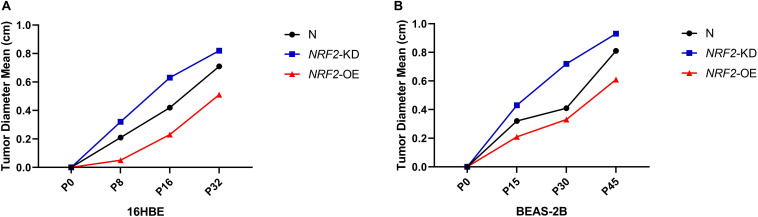
NRF2 expression affects the tumorigenicity of Nano-SiO_2_-transformed cells in nude mice. **(A,B)** The major diameter of the tumor formed at the subcutaneous injection site in nude mice. Tumor sizes were measured precisely 5 weeks after injection. The data are presented as a mean of tumor diameter. N: 16HBE or BEAS-2B cells without transfection; NRF2-KD: knockdown of NRF2; NRF2-OE: overexpression of NRF2.

Since the expression of the NRF2 gene is up-regulated by Nano-SiO_2_, we further investigated the role of NRF2 in carcinogenesis induced by Nano-SiO_2_ by knockdown or overexpression of the NRF2 gene. Cells were transfected with vector harboring the NRF2 gene or the NRF2 gene shRNA to achieve overexpression (NRF2-OE) or knockdown (NRF2-KD) of the NRF2 gene and levels of NRF2 protein were determined using western blot (Supplementary Figures S1A–D). Cell viability assay showed that neither knockdown nor overexpression of the NRF2 gene introduces cytotoxicity to 16HBE and BEAS-2B cells (Supplementary Figures S1E,F). Thereafter, the effects of NRF2-OE and NRF2-KD on the viability of cells treated with Nano-SiO_2_ were determined using the MTT method. We observed that NRF2-OE increases the viability of both 16HBE and BEAS-2B cells after treated with Nano-SiO_2_, whereas NRF2-KD tends to reduce the cell viability upon Nano-SiO_2_ treatment (Figure 7). These results suggest that the NRF2 attenuates the cytotoxicity induced by Nano-SiO_2_ exposure. To investigate the role of NRF2 protein in the tumorigenicity induced by Nano-SiO_2_, the tumorigenicity of cells with NRF2-OE or NRF2-KD exposed to Nano-SiO_2_ were determined both *in vitro* and *in vivo*. The 16HBE and BEAS-2B cells with NRF2-OE or NRF2-KD were treated with Nano-SiO_2_ (10.0 μg/ml for 16HBE cells, 40.0 μg/ml for BEAS-2B cells) for various passages and followed with soft agar colony formation assay and nude mice injection. With the prolonged Nano-SiO_2_ exposure, both NRF2-OE and NRF2-KD cells (16HBE and BEAS-2B) developed a significant amount of colonies in the soft agar (Tables 2, 3). Interestingly, we observed that cells with NRF2-OE formed a lower number of colonies than cells of the control group, while NRF2-KD was able to increase the formation of colonies on soft agar. Consistent with the *in vitro* assay, both 16HBE (P8, P16, P32) and BEAS-2B (P15, P30, P45) cells with NRF2-OE formed tumors of a smaller size than the control group, while cells with NRF2-KD tend to increase the average tumor size (Figure 8). Taken together, results from both the *in vitro* and *in vivo* assay suggest that the NRF2 protein plays an inhibitory role in the tumorigenesis induced by Nano-SiO_2_ exposure.

**TABLE 2:** Summary of colony formation in soft agar of 16HBE cells treated with Nano-SiO_2_.

**Treatment Group**	**Cell number**	**Number of Colonies**		
		**16HBE**	**16HBE-ShRNA-NRF2**	**16HBE-pEGFP-NRF2**
P0	1 × 10^4^	273 ± 8	284 ± 12	267 ± 10
P8	1 × 10^4^	555 ± 32*	730 ± 30^*#^	498 ± 13^*#^
P16	1 × 10^4^	1141 ± 73*	1758 ± 108^*#^	846 ± 47^*#^
P32	1 × 10^4^	2161 ± 150*	2247 ± 64^*#^	1486 ± 43^*#^

* p < 0.05, compared with normal cells without Nano-SiO_2_ exposure; ^#^ p < 0.05, compared with normal cells treated with Nano-SiO_2_ for the same passages.

**TABLE 3:** Summary of colony formation in soft agar of BEAS-2B cells treated with Nano-SiO_2_.

**Treatment Group**	**Cell numbers**	**Number of Colonies**		
		**BEAS-2B**	**BEAS-2B-ShRNA-NRF2**	**BEAS-2B-pEGFP-NRF2**
P0	1 × 10^4^	254 ± 14	260 ± 8	246 ± 7
P15	1 × 10^4^	440 ± 14*	844 ± 53^*#^	324 ± 19^*#^
P30	1 × 10^4^	996 ± 80*	1476 ± 51^*#^	705 ± 72^*#^
P45	1 × 10^4^	1788 ± 69*	1915 ± 35^*#^	1155 ± 79^*#^

*p < 0.05, compared with normal cells without Nano-SiO_2_ exposure; ^#^*p* < 0.05, compared with normal cells treated with Nano-SiO_2_ for the same passages.

## Discussion

In this study, two types of human bronchial epithelial cell lines (16HBE and BEAS-2B) were selected as the *in vitro* models to investigate the carcinogenicity of Nano-SiO_2_. We observed the dose-dependent cytotoxic effects of Nano-SiO_2_ on the human bronchial epithelial cells. Also, we showed the tumorigenicity of Nano-SiO_2_ in human bronchial epithelial cells.

As a hallmark of carcinogenesis, alterations in DNA methylation occur frequently in a wide range of cancers (Miousse et al., 2018; Dreval et al., 2019). Our results demonstrated a global genomic hypomethylation in cells exposed to the Nano-SiO_2_, which is consistent with the reduction of the total enzyme activity of DNA methyltransferases. Although the total activity of DNMTs is reduced, we found that the expression of DNMT1 protein is elevated and the expression of DNMT3a protein is downregulated. The DNMT1 is responsible for the methylation of tumor suppressor genes (Jair et al., 2006; Ting et al., 2006), the increased expression of which might induce hypermethylation of the tumor suppressor genes, thereby inhibiting the expression of tumor suppressors and promoting carcinogenesis induced by Nano-SiO_2_. The DNMT3a protein is known for the *de novo* CpG methylation independent of replication, which is consistent with the global hypomethylation induced by Nano-SiO_2_. Loss of DNMT3a has been reported to be associated with leukemia pathogenesis and poor prognosis (Shivarov et al., 2013). Moreover, MBD2 and MeCP2 that can bind to and mediate the repression of methylated tumor suppressor genes (Klose and Bird, 2006; Lopez-Serra et al., 2008; Mian et al., 2011), were also induced by the Nano-SiO_2_ exposure.

In addition to the global DNA hypomethylation, we observed a decreased methylation of CpG islands within the NRF2 promoter region in human bronchial epithelial cells exposed to Nano-SiO_2_. The NRF2 protein is a key transcription factor involved in cellular defensive mechanisms against stresses induced by electrophiles, oxidants, and chemicals (Motohashi and Yamamoto, 2004). Under the basal condition, NRF2 is localized in the cytoplasm by binding to its cytosolic repressor Kelch-like ECH-associated protein 1 (KEAP1) (Motohashi and Yamamoto, 2004). In response to stress, NRF2 dissociates from KEAP1 and translocates to the nucleus, where it activates the transcription of its target genes to maintain cellular homeostasis (Panieri and Saso, 2019). Short-term exposure to Nano-SiO_2_ can increase the expression of the NRF2 gene (Liu et al., 2017), which is consistent with our results that the NRF2 gene expression is up-regulated in both mRNA and protein levels in cells exposed to Nano-SiO_2_ exposure. Moreover, our findings revealed that levels of HO1, SOD1, and GST, which are encoded by antioxidative gene targets of the NRF2 protein (Itoh et al., 1997), are increased upon Nano-SiO_2_ exposure. These results suggest that NRF2 promotes the stress responses of cells treated with Nano-SiO_2_.

Activation of the NRF2 protein is suggested to suppress carcinogenesis, especially in its early stage (Wu et al., 2019). Loss of NRF2 disrupts the antioxidant axis resulting in increased oxidative stress, ultimately leading to DNA damage and the initiation of malignant cellular transformation (Frohlich et al., 2008). In the tumor microenvironment, the tumor suppressor gene BRCA1 activates NRF2 (Gorrini et al., 2013), whereas the oncogene *Fyn* mediates nuclear export and induces degradation of NRF2 (Niture et al., 2014). NRF2-deficient mice are more susceptible to carcinogens and develop severer tumors in the urinary bladder and liver (Iida et al., 2007; Kitamura et al., 2007).

Collectively, our results showed that NRF2 plays an important role in protecting against the carcinogenesis induced by Nano-SiO_2_ exposure. Moreover, hypomethylation at the promoter region of the NRF2 gene contributed to the alterations of NRF2 upon Nano-SiO_2_ exposure. It should, however, be noted that histone modifications are also important for NRF2 gene transcription and it cannot be excluded that the alterations of NRF2 at both transcription and protein levels are caused by a more intrinsic mechanism.

## Data Availability Statement

The raw data supporting the conclusions of this article will be made available by the authors, without undue reservation.

## Ethics Statement

The animal study was reviewed and approved by Shanghai Municipal Center for Disease Prevention and Control.

## Author Contributions

DL, GT, and YS jointly conceived this project and supervised the experiments. DL and XW designed the research. DL, XW, PX, QH, JS, and XH performed the experiments and analyzed the experimental results. DL, XW, YS, and GT prepared the manuscript. All authors contributed to the article and approved the submitted version.

## Conflict of Interest

YS is employed by the Syngenta (China) Investment Company Limited. The remaining authors declare that the research was conducted in the absence of any commercial or financial relationships that could be construed as a potential conflict of interest.

## References

[B1] Bermudez E. Mangum J. B. Wong B. A. Asgharian B. Hext P. M. Warheit D. B. (2004). Pulmonary responses of mice, rats, and hamsters to subchronic inhalation of ultrafine titanium dioxide particles. *Toxicol. Sci.* 77 347–357. 10.1093/toxsci/kfh019 14600271

[B2] Chen M. von Mikecz A. (2005). Formation of nucleoplasmic protein aggregates impairs nuclear function in response to SiO2 nanoparticles. *Exp. Cell Res.* 305 51–62. 10.1016/j.yexcr.2004.12.021 15777787

[B3] Dreval K. Tryndyak V. De Conti A. Beland F. A. Pogribny I. P. (2019). Gene expression and DNA methylation alterations during non-alcoholic steatohepatitis-associated liver carcinogenesis. *Front. Genet.* 10:486. 10.3389/fgene.2019.00486 31191608 PMC6549534

[B4] Frohlich D. A. Mccabe M. T. Arnold R. S. Day M. L. (2008). The role of Nrf2 in increased reactive oxygen species and DNA damage in prostate tumorigenesis. *Oncogene* 27 4353–4362. 10.1038/onc.2008.79 18372916

[B5] Fruijtier-Polloth C. (2012). The toxicological mode of action and the safety of synthetic amorphous silica-a nanostructured material. *Toxicology* 294 61–79. 10.1016/j.tox.2012.02.001 22349641

[B6] Gama-Sosa M. A. Slagel V. A. Trewyn R. W. Oxenhandler R. Kuo K. C. Gehrke C. W. (1983). The 5-methylcytosine content of DNA from human tumors. *Nucleic Acids Res.* 11 6883–6894. 10.1093/nar/11.19.6883 6314264 PMC326421

[B7] Gebel T. (2012). Small difference in carcinogenic potency between GBP nanomaterials and GBP micromaterials. *Arch. Toxicol.* 86 995–1007. 10.1007/s00204-012-0835-1 22418597

[B8] Gong C. Tao G. Yang L. Liu J. He H. Zhuang Z. (2012a). The role of reactive oxygen species in silicon dioxide nanoparticle-induced cytotoxicity and DNA damage in HaCaT cells. *Mol. Biol. Rep.* 39 4915–4925. 10.1007/s11033-011-1287-z 22179747

[B9] Gong C. Tao G. Yang L. Liu J. Liu Q. Li W. (2012b). Methylation of PARP-1 promoter involved in the regulation of nano-SiO2-induced decrease of PARP-1 mRNA expression. *Toxicol. Lett.* 209 264–269. 10.1016/j.toxlet.2012.01.007 22265868

[B10] Gong C. Tao G. Yang L. Liu J. Liu Q. Zhuang Z. (2010). SiO(2) nanoparticles induce global genomic hypomethylation in HaCaT cells. *Biochem. Biophys. Res. Commun.* 397 397–400. 10.1016/j.bbrc.2010.05.076 20501321

[B11] Gorrini C. Baniasadi P. S. Harris I. S. Silvester J. Inoue S. Snow B. (2013). BRCA1 interacts with Nrf2 to regulate antioxidant signaling and cell survival. *J. Exp. Med.* 210 1529–1544. 10.1084/jem.20121337 23857982 PMC3727320

[B12] Hirose A. Takagi A. Nishimura T. Tsuda H. Sakamoto Y. Ogata A. (2011). [Importance of researches on chronic effects by manufactured nanomaterials]. *Yakugaku Zasshi* 131 195–201. 10.1248/yakushi.131.195 21297361

[B13] Iarc. (1997). Silica, some silicates, coal dust and para-aramid fibrils. *IARC Monogr. Eval. Carcinog. Risks Hum.* 68 1–475. 9303953 PMC5366849

[B14] Iida K. Itoh K. Maher J. M. Kumagai Y. Oyasu R. Mori Y. (2007). Nrf2 and p53 cooperatively protect against BBN-induced urinary bladder carcinogenesis. *Carcinogenesis* 28 2398–2403. 10.1093/carcin/bgm146 17602169

[B15] Itoh K. Chiba T. Takahashi S. Ishii T. Igarashi K. Katoh Y. (1997). An Nrf2/small Maf heterodimer mediates the induction of phase II detoxifying enzyme genes through antioxidant response elements. *Biochem. Biophys. Res. Commun.* 236 313–322. 10.1006/bbrc.1997.6943 9240432

[B16] Jair K. W. Bachman K. E. Suzuki H. Ting A. H. Rhee I. Yen R. W. (2006). De novo CpG island methylation in human cancer cells. *Cancer Res.* 66 682–692. 10.1158/0008-5472.can-05-1980 16423997

[B17] Kang K. A. Piao M. J. Kim K. C. Kang H. K. Chang W. Y. Park I. C. (2014). Epigenetic modification of Nrf2 in 5-fluorouracil-resistant colon cancer cells: involvement of TET-dependent DNA demethylation. *Cell Death Dis.* 5:e1183. 10.1038/cddis.2014.149 24743738 PMC4001304

[B18] Kasai T. Umeda Y. Ohnishi M. Mine T. Kondo H. Takeuchi T. (2016). Lung carcinogenicity of inhaled multi-walled carbon nanotube in rats. *Part Fibre Toxicol.* 13:53. 10.1186/s12989-016-0164-2 PMC5064785 27737701

[B19] Khor T. O. Fuentes F. Shu L. Paredes-Gonzalez X. Yang A. Y. Liu Y. (2014). Epigenetic DNA methylation of antioxidative stress regulator NRF2 in human prostate cancer. *Cancer Prev. Res. (Phila)* 7 1186–1197. 10.1158/1940-6207.capr-14-0127 25266896 PMC4256109

[B20] Kitamura Y. Umemura T. Kanki K. Kodama Y. Kitamoto S. Saito K. (2007). Increased susceptibility to hepatocarcinogenicity of Nrf2-deficient mice exposed to 2-amino-3-methylimidazo[4,5-f]quinoline. *Cancer Sci.* 98 19–24. 10.1111/j.1349-7006.2006.00352.x 17083568 PMC11159668

[B21] Klose R. J. Bird A. P. (2006). Genomic DNA methylation: the mark and its mediators. *Trends Biochem. Sci.* 31 89–97. 10.1016/j.tibs.2005.12.008 16403636

[B22] Liu D. Zhang Y. Wei Y. Liu G. Liu Y. Gao Q. (2016). Activation of AKT pathway by Nrf2/PDGFA feedback loop contributes to HCC progression. *Oncotarget* 7 65389–65402. 10.18632/oncotarget.11700 27588483 PMC5323163

[B23] Liu W. Hu T. Zhou L. Wu D. Huang X. Ren X. (2017). Nrf2 protects against oxidative stress induced by SiO2 nanoparticles. *Nanomedicine (Lond)* 12 2303–2318. 10.2217/nnm-2017-0046 28952419

[B24] Lopez-Serra L. Ballestar E. Ropero S. Setien F. Billard L. M. Fraga M. F. (2008). Unmasking of epigenetically silenced candidate tumor suppressor genes by removal of methyl-CpG-binding domain proteins. *Oncogene* 27 3556–3566. 10.1038/sj.onc.1211022 18223687

[B25] Lu K. Alcivar A. L. Ma J. Foo T. K. Zywea S. Mahdi A. (2017). NRF2 induction supporting breast cancer cell survival is enabled by oxidative stress-induced DPP3-KEAP1 interaction. *Cancer Res.* 77 2881–2892. 10.1158/0008-5472.can-16-2204 28416489 PMC5464605

[B26] Lu X. Jin T. Jin Y. Wu L. Hu B. Tian Y. (2013). Toxicogenomic analysis of the particle dose- and size-response relationship of silica particles-induced toxicity in mice. *Nanotechnology* 24 015106 10.1088/0957-4484/24/1/015106 23221170

[B27] Marhenke S. Lamle J. Buitrago-Molina L. E. Canon J. M. Geffers R. Finegold M. (2008). Activation of nuclear factor E2-related factor 2 in hereditary tyrosinemia type 1 and its role in survival and tumor development. *Hepatology* 48 487–496. 10.1002/hep.22391 18666252

[B28] Mian O. Y. Wang S. Z. Zhu S. Z. Gnanapragasam M. N. Graham L. Bear H. D. (2011). Methyl-binding domain protein 2-dependent proliferation and survival of breast cancer cells. *Mol. Cancer Res.* 9 1152–1162. 10.1158/1541-7786.mcr-11-0252 21693597 PMC3157569

[B29] Miousse I. R. Ewing L. E. Kutanzi K. R. Griffin R. J. Koturbash I. (2018). DNA methylation in radiation-induced carcinogenesis: experimental evidence and clinical perspectives. *Crit. Rev. Oncogen.* 23 1–11. 10.1615/critrevoncog.2018025687 29953365 PMC6369919

[B30] Mohajerani A. Burnett L. Smith J. V. Kurmus H. Milas J. Arulrajah A. (2019). Nanoparticles in construction materials and other applications, and implications of nanoparticle use. *Materials (Basel)* 12 3052. 10.3390/ma12193052 31547011 PMC6804222

[B31] Motohashi H. Yamamoto M. (2004). Nrf2-Keap1 defines a physiologically important stress response mechanism. *Trends Mol. Med.* 10 549–557. 10.1016/j.molmed.2004.09.003 15519281

[B32] Napierska D. Thomassen L. C. Lison D. Martens J. A. Hoet P. H. (2010). The nanosilica hazard: another variable entity. *Part Fibre Toxicol.* 7:39. 10.1186/1743-8977-7-39 21126379 PMC3014868

[B33] Niture S. K. Khatri R. Jaiswal A. K. (2014). Regulation of Nrf2-an update. *Free Radic. Biol. Med.* 66 36–44. 10.1016/j.freeradbiomed.2013.02.008 23434765 PMC3773280

[B34] Panieri E. Saso L. (2019). Potential applications of NRF2 inhibitors in cancer therapy. *Oxid. Med. Cell Longev.* 2019:8592348. 10.1155/2019/8592348 PMC6487091 31097977

[B35] Seidel C. Kirsch A. Fontana C. Visvikis A. Remy A. Gate L. (2017). Epigenetic changes in the early stage of silica-induced cell transformation. *Nanotoxicology* 11 923–935. 10.1080/17435390.2017.1382599 28958182

[B36] Shivarov V. Gueorguieva R. Stoimenov A. Tiu R. (2013). DNMT3A mutation is a poor prognosis biomarker in AML: results of a meta-analysis of 4500 AML patients. *Leuk Res.* 37 1445–1450. 10.1016/j.leukres.2013.07.032 23962568

[B37] Song Y. Li X. Du X. (2009). Exposure to nanoparticles is related to pleural effusion, pulmonary fibrosis and granuloma. *Eur. Respir. J.* 34 559–567. 10.1183/09031936.00178308 19696157

[B38] Sweeney S. K. Luo Y. O’donnell M. A. Assouline J. (2016). Nanotechnology and cancer: improving real-time monitoring and staging of bladder cancer with multimodal mesoporous silica nanoparticles. *Cancer Nanotechnol.* 7:3. 10.1186/s12645-016-0015-8 PMC4846680 27217840

[B39] Ting A. H. Jair K. W. Schuebel K. E. Baylin S. B. (2006). Differential requirement for DNA methyltransferase 1 in maintaining human cancer cell gene promoter hypermethylation. *Cancer Res.* 66 729–735. 10.1158/0008-5472.can-05-1537 16424002

[B40] Vivero-Escoto J. L. Huxford-Phillips R. C. Lin W. (2012). Silica-based nanoprobes for biomedical imaging and theranostic applications. *Chem. Soc. Rev* 41 2673–2685. 22234515 10.1039/c2cs15229k PMC3777230

[B41] Wang J. J. Sanderson B. J. Wang H. (2007). Cytotoxicity and genotoxicity of ultrafine crystalline SiO2 particulate in cultured human lymphoblastoid cells. *Environ. Mol. Mutagen.* 48 151–157. 10.1002/em.20287 17285640

[B42] Wu S. Lu H. Bai Y. (2019). Nrf2 in cancers: a double-edged sword. *Cancer Med.* 8 2252–2267. 10.1002/cam4.2101 30929309 PMC6536957

[B43] Yu S. Khor T. O. Cheung K. L. Li W. Wu T. Y. Huang Y. (2010). Nrf2 expression is regulated by epigenetic mechanisms in prostate cancer of TRAMP mice. *PLoS ONE* 5:e8579. 10.1371/journal.pone.0008579 20062804 PMC2799519

[B44] Yu Y. Duan J. Li Y. Yu Y. Jin M. Li C. (2015). Combined toxicity of amorphous silica nanoparticles and methylmercury to human lung epithelial cells. *Ecotoxicol. Environ. Saf.* 112 144–152. 10.1016/j.ecoenv.2014.10.026 25463865

[B45] Zhang C. Wang H. J. Bao Q. C. Wang L. Guo T. K. Chen W. L. (2016). NRF2 promotes breast cancer cell proliferation and metastasis by increasing RhoA/ROCK pathway signal transduction. *Oncotarget* 7 73593–73606. 10.18632/oncotarget.12435 27713154 PMC5342001

[B46] Zhao X. Q. Zhang Y. F. Xia Y. F. Zhou Z. M. Cao Y. Q. (2015). Promoter demethylation of nuclear factor-erythroid 2-related factor 2 gene in drug-resistant colon cancer cells. *Oncol. Lett.* 10 1287–1292. 10.3892/ol.2015.3468 26622665 PMC4533726

