# Supplementary material for: Demethylation of the NRF2 Promoter Protects Against Carcinogenesis Induced by Nano-SiO2
Source: Front Genet. 2020 Jul 28;11:818. doi: 10.3389/fgene.2020.00818 (PMC7399247; doi:10.3389/fgene.2020.00818)
Supplement: Supplementary file 1 [file Data_Sheet_1.docx]

Table S1. List of primers information

| Gene | Sequence (5’-3’) |
| --- | --- |
| NRF2 | F: ACGGTATGCAACAGGACATTGAGC |
|  | R: TTGGCTTCTGGACTTGGAACCATG |
| HO1 | F: CCTCCCTGTACCACATCTATGT |
|  | R: GCTCTTCTGGGAAGTAGACAG |
| SOD1 | F: ATCCTCTATCCAGAAAACACGG |
|  | R: GCGTTTCCTGTCTTTGTACTTT |
| GST | F: TCTCCAGATTCCCATCCACTTCCC |
|  | R: CTGCGGCTCGGTGATGTCTTC |
| NRF2_M1 | F: TTATTATGATGGATTTGGAGTTGTC |
|  | R: CTAACCAAACGTAAAAAAAACCG |
| NRF2_U1 | F: ATTATGATGGATTTGGAGTTGTTGT |
|  | R: CTAACCAAACATAAAAAAAACCAAT |
| NRF2_M2 | F: TGTCGGTAAAAATGTGTTTAGTTAC |
|  | R: CTCAAAACTACCAAAAAATAATCCG |
| NRF2_U2 | F: TTGGTAAAAATGTGTTTAGTTATGG |
|  | R: AAAACTACCAAAAAATAATCCAAA |


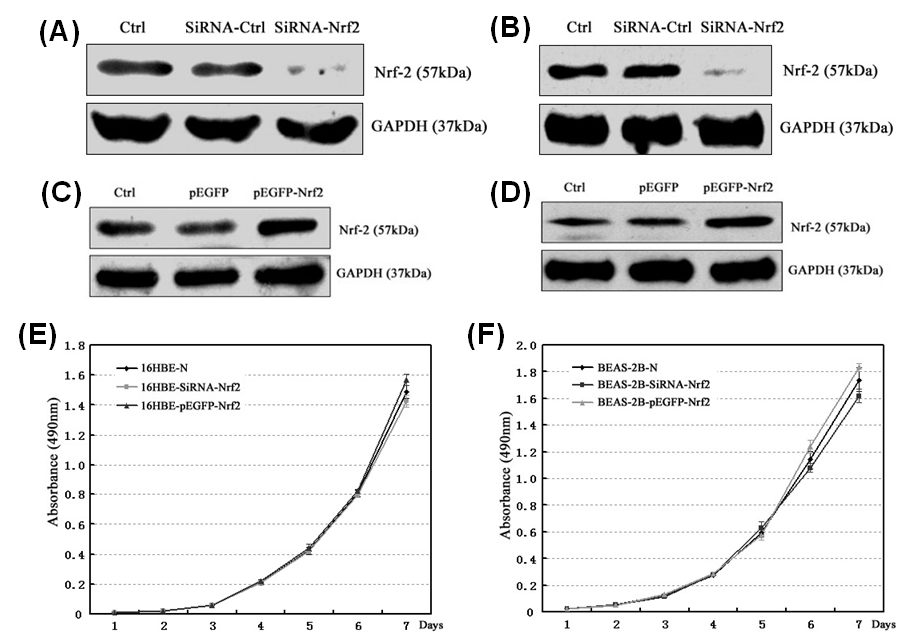


Figure S1. Knockdown and overexpression of *NRF2* in human bronchial epithelial cells. Expression of NRF2 protein was identified in 16HBE cells (A and C) and BEAS-2B cells (B and D) by Western blotting. GAPDH was used as the internal control for normalization. (E-F) Line plots showing that either knockdown or overexpression of *NRF2* has no effects on cell growth curve when compared with the normal cells (16HBE-N or BEAS-2B-N).
